# Recurrence of Endometrial Stromal Sarcoma, Two Decades Post-Treatment

**DOI:** 10.7759/cureus.9249

**Published:** 2020-07-17

**Authors:** Mounika Gangireddy, Janet Chan Gomez, Tejaswi Kanderi, Maria Joseph, Vishwa Kundoor

**Affiliations:** 1 Internal Medicine, University of Pittsburgh Medical Center Pinnacle, Harrisburg, USA; 2 Internal Medicine, MedStar Franklin Square Medical Center, Baltimore, USA

**Keywords:** endometrial stromal cell sarcoma, oncology, gynecology, uterine sarcoma, recurrent uterine sarcoma, uterine malignancy

## Abstract

Endometrial stromal cell sarcomas (ESS) are a unique subtype of uterine malignancy. Recurrent low grade endometrial stromal sarcomas (LESS) is identified in half of the patients. Here, we discuss a case of a 76-year-old Asian female with a past medical history of adenomyosis and hypertension who presented to the outpatient clinic with a chief complaint of painless hematuria for one day. Computed tomography scan of abdomen and pelvis with contrast showed a new right-sided mixed cystic and solid pelvic mass measuring up to 6 cm, obstructing and invading the distal right ureter, which was concerning for malignancy. Positron emission tomography (PET scan) demonstrated a right pelvic mass with increased radiotracer activity consistent with malignancy. She underwent laparotomy with excision of the right-sided pelvic mass with an abdominal washout and at the same time, also underwent cystoscopy with right ureteral stent placement. Tissue pathology was consistent with spindle cell neoplasm with staining and histologic features consistent with a recurrent stromal cell sarcoma. Uterine sarcomas tend to have an aggressive nature but there are key features about ESS that distinguish it from other uterine sarcomas. ESS has a more indolent clinical course and can reoccur years after initial diagnosis. They usually relapse locally, although relapses in extra-uterine sites have also been reported. Treatment of ESS depends on the grade and stage at the time of diagnosis. The main line of treatment for ESS consists of a total abdominal hysterectomy (TAH) and salpingo-oophorectomy (BSO). The significance of this case demonstrates that, although remission can be obtained after the initial diagnosis, recurrence can happen. Even when patients seem to be disease-free, clinicians should follow them closely; early diagnosis is important as treatment for this type of entity has a high survival rate.

## Introduction

Uterine sarcomas are an uncommon type of malignant mesenchymal tumor. They only account for 1% of all gynecological malignancies and 4-9% of all malignant uterine neoplasms [1-2]. The incidence of uterine sarcomas is one to two cases per 100,000 per year in the general population [3-4]. Endometrial stromal cell sarcoma (ESS) is a rare and indolent type of uterine tumor. ESS account for 7-25% of all uterine mesenchymal tumors and <1% of all uterine tumors [3-5]. ESS is most frequently seen in premenopausal women, affecting between 40-55 years, although it can occur in older women [1,3-4,6]. Low grade endometrial stromal sarcomas (LESS) is a type of ESS. Recurrent LESS is identified in half of the patients [7]. At the time of reappearance, it is mostly limited to the pelvis but it can invade the urinary tract , and in rare instances, the lung [7]. Here, we describe a case of recurrent endometrial stromal cell sarcoma more than two decades after initial treatment.

## Case presentation

A 76-year-old Asian female patient with a past medical history of hypertension and adenomyosis presented to the outpatient clinic with a chief complaint of painless hematuria for one day, which was associated with generalized weakness for a few weeks prior to presentation. She denied weight loss, nausea, diarrhea, or abdominal pain. The remainder of the review of symptoms was negative. On physical exam, she was afebrile, with a respiratory rate of 15 per minute, heart rate of 80 beats per minute, blood pressure of 130/90 millimeter of mercury, and saturating 99% on room air. Physical examination revealed a mobile mass in the right lower quadrant. She was subsequently referred to the emergency department. The laboratory results on admission are revealed in Table 1.

**Table 1:** Laboratory results on admission

Lab	Value	Reference Range
White blood cell (K/uL)	6.5	3.9-9.5
Hemoglobin (g/dL)	13.6	11.7-15.1
Hematocrit (%)	40.5	29.4-47
Platelet (K/uL)	138	144-366
Blood urea nitrogen(mg/dL)	14	7-25
Creatinine(mg/dL)	1.1	0.6-1.2

**Figure 1:**
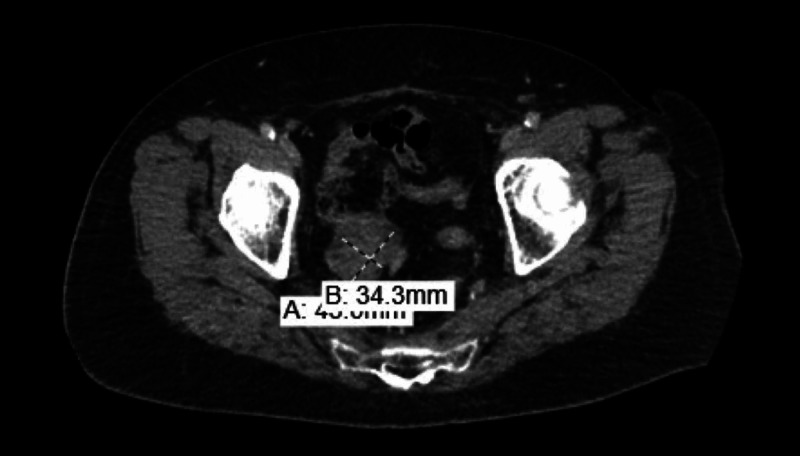
Sagittal view of CT abdomen and pelvis reveals a large solid/cystic pelvic mass surrounding the right ureter

**Figure 2:**
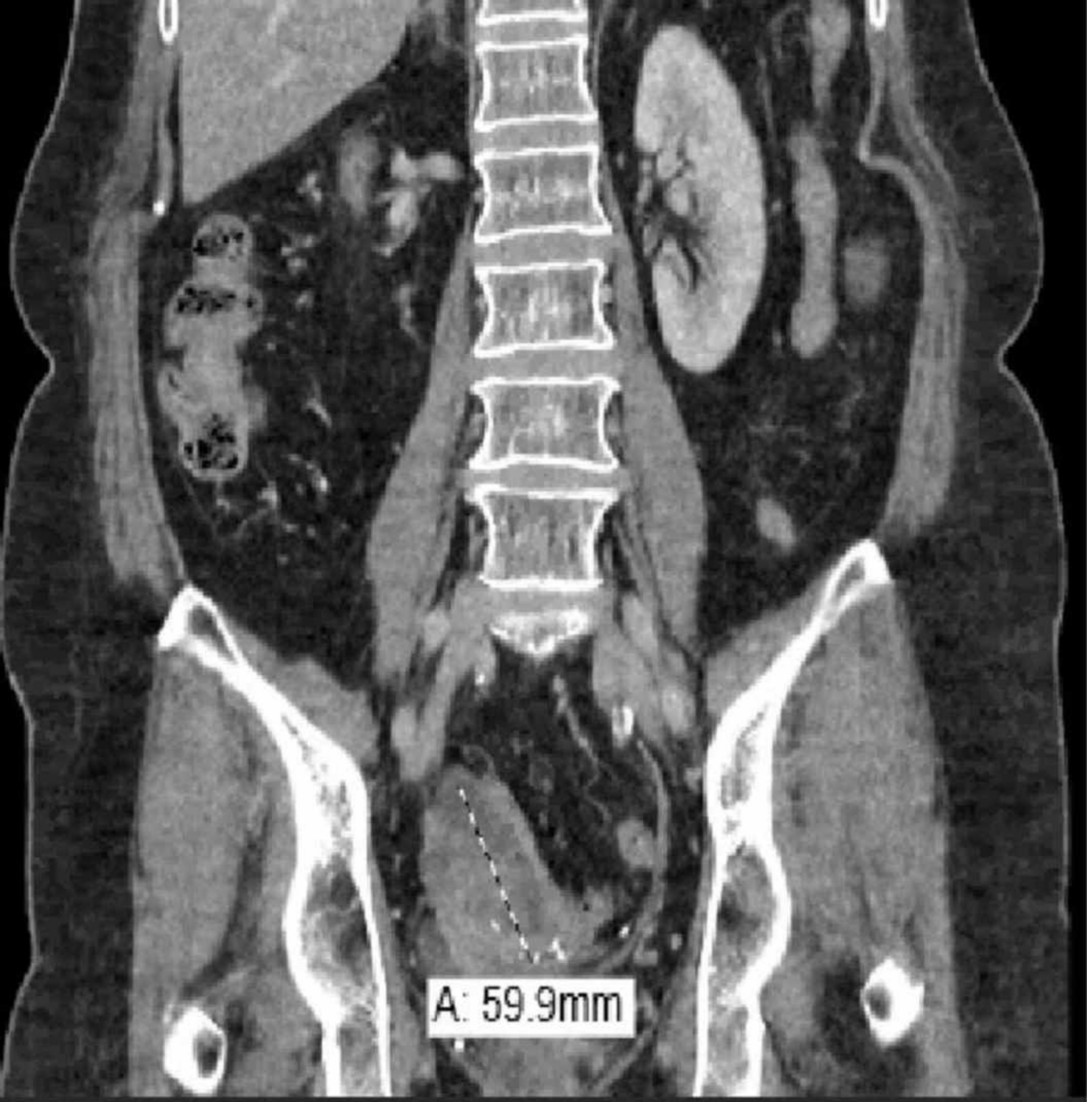
Coronal view of CT abdomen and pelvis reveals a large solid/cystic pelvic mass surrounding the right ureter

CT scans of abdomen/pelvis with IV contrast showed new right-sided mixed cystic and solid pelvic mass measuring up to 6 cm, which obstructed and invaded the distal right ureter, concerning for malignancy (Figures 1-2).

 CT chest with IV contrast showed multiple pulmonary nodules less than 5 mm. PET/CT revealed increased radiotracer activity in the right pelvic mass consistent with malignancy. Tumor markers including CA-125, CEA, and CA 27-29 were negative. Colonoscopy, mammogram as, well as a recent pap smear, were all negative for malignancy. Based on the diagnostic workup, the patient was thought to have a relapse of sarcoma. She underwent laparotomy with excision of the right-sided pelvic mass along with an abdominal washout. At the same time, she also underwent cystoscopy with right ureteral stent placement.

**Figure 3:**
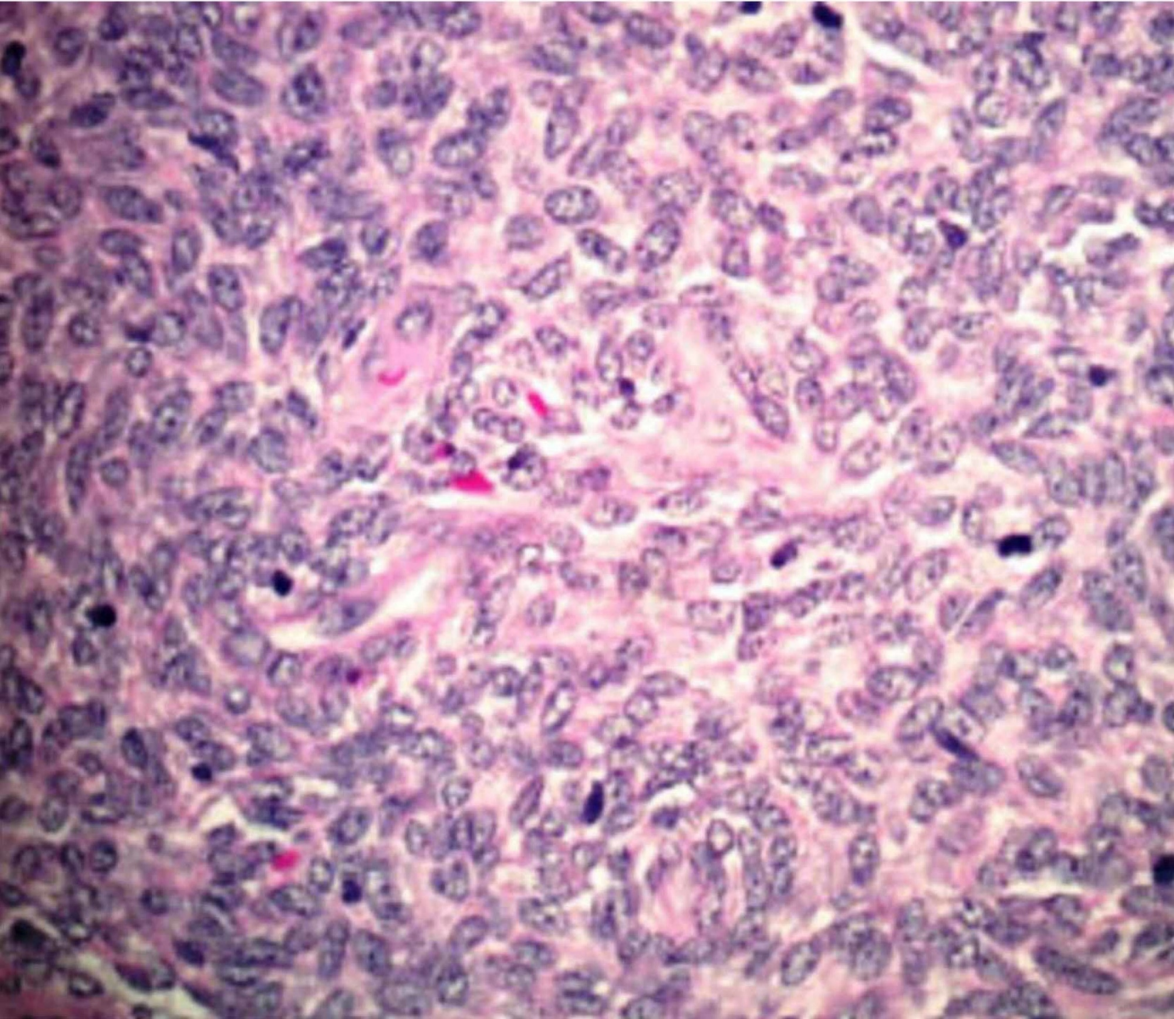
Tissue specimen showing proliferating spindle cells with mild atypia

Tissue pathology was consistent with spindle cell neoplasm and histologic features were consistent with a recurrent stromal cell sarcoma as shown in Figure 3. The microscopic description of the mass revealed a spindled blue cell lesion with no necrosis, but a moderate mitotic rate. Immunohistochemistry was strongly positive for estrogen receptor (ER), progesterone receptor (PR), WT-1, CD99, and CD 56. There was focal weak staining with synaptophysin and CD 10. It was negative for inhibin, chromogranin, calretinin, cytokeratin 7, CD 34, BCL1, MSA, and SMA.

Prior to the current presentation, the patient had been diagnosed with stage 1B low-grade endometrial stromal cell carcinoma (as per the FIGO [Federation of Gynecology and Obstetrcs] staging) when she was 50 years old. Back then, she presented with abnormal uterine bleeding and CT scans of her abdomen and pelvis showed an 11 cm uterine mass. She underwent total abdominal hysterectomy with bilateral salpingo-oophorectomy, without adjuvant therapy. She was under surveillance every six months for the first two years, then annually after for the next 10 years post-surgery. 

Tissue pathology biopsy findings were similar to the findings found two decades ago, indicating the recurrence of endometrial stromal cell sarcoma. Repeat CT abdomen and pelvis after the surgery showed a diminished right-sided pelvic mass. She was classified as endometrial stromal cell cancer with local recurrence. She did not receive adjuvant therapy. She is currently under surveillance every six months and has been doing well in the last two years. 

## Discussion

Uterine sarcomas can be classified into four histological entities: carcinosarcomas (40% of cases), leiomyosarcoma (40% of cases), endometrial stromal sarcomas (10-15% of cases) and undifferentiated or “other” sarcomas (5-10% of cases) [8]. The focus of this paper will be endometrial stromal sarcoma (ESS). Histopathology of ESS is characteristic of spindle cell proliferation resembling normal endometrial stromal cells in the proliferative phase [6]. ESS can be divided into three clinicopathological entities: endometrial stromal nodules (ESN), undifferentiated endometrial stromal sarcomas (UES), and LESS [6,9]. ESN does not invade the myometrium [5]. Meanwhile, LESS and UES have myometrial and vascular invasion [5]. 

Various risk factors may contribute to developing ESS, such as increasing age, polycystic ovarian disease, diabetes, obesity, adenomyosis, endometriosis, family history of endometrial cancer, tamoxifen use, estrogen therapy, history of pelvic radiation and history of breast or ovarian cancer [7-10]. Our patient did have a history of adenomyosis, which might have increased her risk of developing ESS.

More than half of the patients are symptomatic at the time of diagnosis, resulting in early diagnosis at an early stage [1,11]. The most common presentation is vaginal bleeding, which is present in 90% of symptomatic patients [6]. Other clinical presentations can include a pelvic or abdominal mass, abdominal distension, pelvic pain or dysmenorrhea but about 25% of patients can be asymptomatic [1,6]. Our patient presented with abnormal uterine bleeding at the time of initial diagnosis but later developed gross hematuria, which is an uncommon presentation for recurrence.

The five-year survival rate for ESS is between 80%-100% [1]. The overall survival depends on the stage. Stage I-II has a five-year survival rate of 90% while stage III-IV has a survival rate of 50% [6,12]. ESS has an indolent course; it may reoccur years after initial diagnosis and usually reoccurs locally, especially in LESS [1]. Our patient had a localized recurrence of LESS confined to pelvis after twenty years post-treatment.

Recurrent ESS

Around 40-50% of patients diagnosed with ESS develop recurrent disease [6-7]. It is often detected many years after a tumor-free interval, as it is a slow-growing tumor [1,7]. The stage at the first time of presentation is the best predictor of reoccurrence risk [1]. The study by Chang et al. revealed the time to recurrence is 65 months in stage I-II and nine months in stage III-IV, there have been cases reported even after this time period [12]. Older age appeared to be the only independent poor prognostic factor for progression-free survival [9]. There are debates on whether, residual ovarian tissue increases the risk of recurrence, especially in earlier stages of ESS [6,9]. Studies have found that specifically in lower stages of ESS, ovarian retention does not impact the overall survival in a completely excised ESS [9]. In our patient, advanced age, lack of adjuvant therapy, and loss of follow up might have resulted in the recurrence of LESS. More studies are needed to determine the risk factors for the recurrence of LESS.

Management

Treatment of ESS depends on the grade and stage at the time of diagnosis. The standard of treatment of ESS for eligible surgical candidates is composed of a total abdominal hysterectomy (TAH) plus salpingo-oophorectomy (BSO) [1-2]. If a patient is not a surgical candidate due to comorbidities and/or high tumor burden such as extensive extra-uterine disease, then a combination of systemic therapy, hormonal therapy, palliative external beam radiation therapy (EBRT) or brachytherapy can be considered [10].

Adjuvant therapies include external beam radiation therapy (EBRT), hormonal therapy, and chemotherapy. Adjuvant EBRT has excellent local control and has delayed recurrences especially in stage II-IV, but has not been shown to improve overall survival [1,12-13]. Many studies have demonstrated that LESS is ER- and PR-positive and adjuvant therapy with hormonal agents can be considered [3,10,14-16]. The hormonal agents frequently used are aromatase inhibitors, fulvestrant, megestrol acetate, and gonadotropin-releasing hormone (GnRH) analogs [10,14,16]. Although adjuvant chemotherapy has been studied in various trials, their effectiveness remains debatable, particularly in advanced and metastatic disease [13]. The most commonly used chemotherapy regimens include doxorubicin and ifosfamide [1-2,10]. 

**Figure 4:**
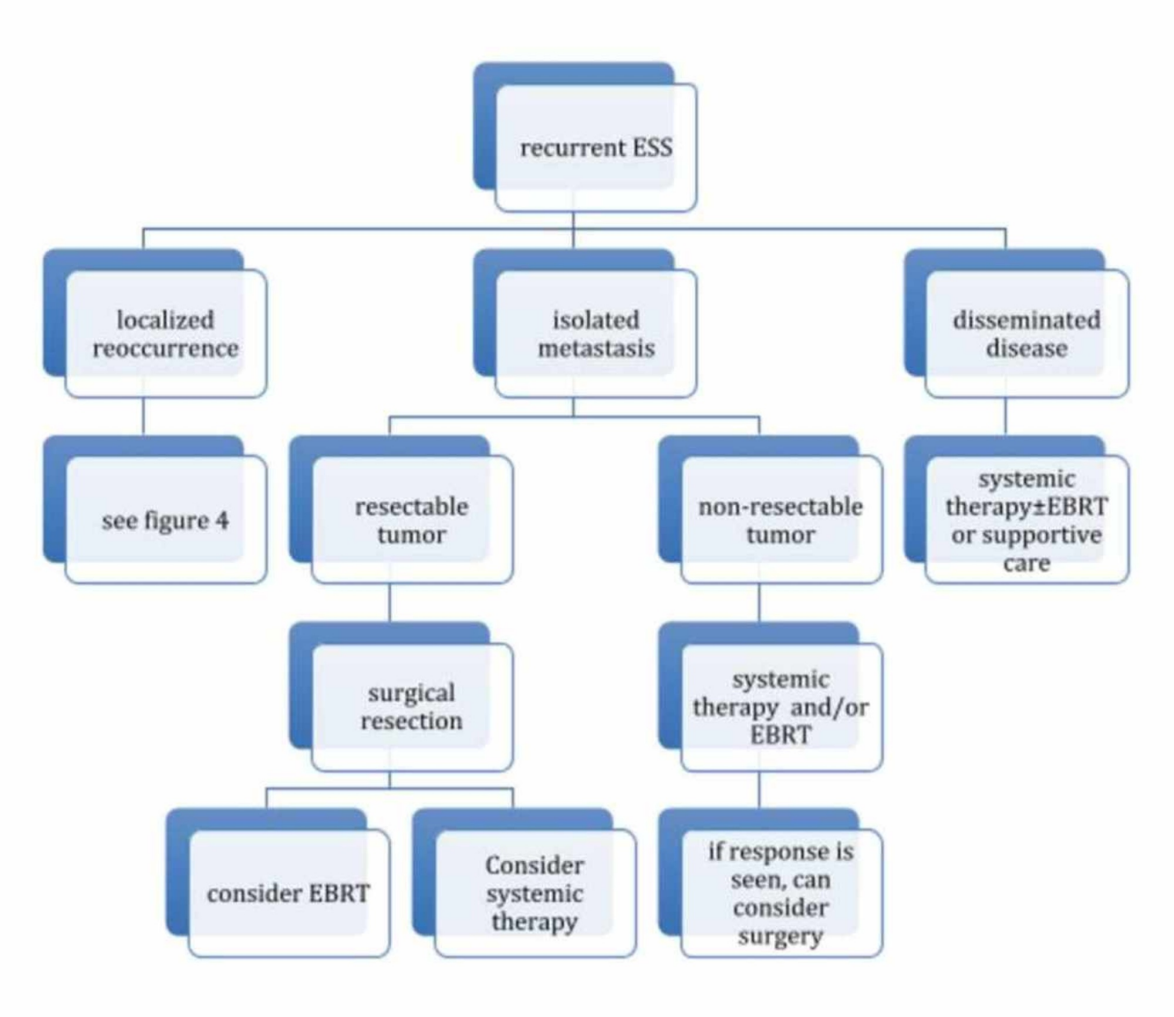
Treatment algorithm for recurrent ESS

**Figure 5:**
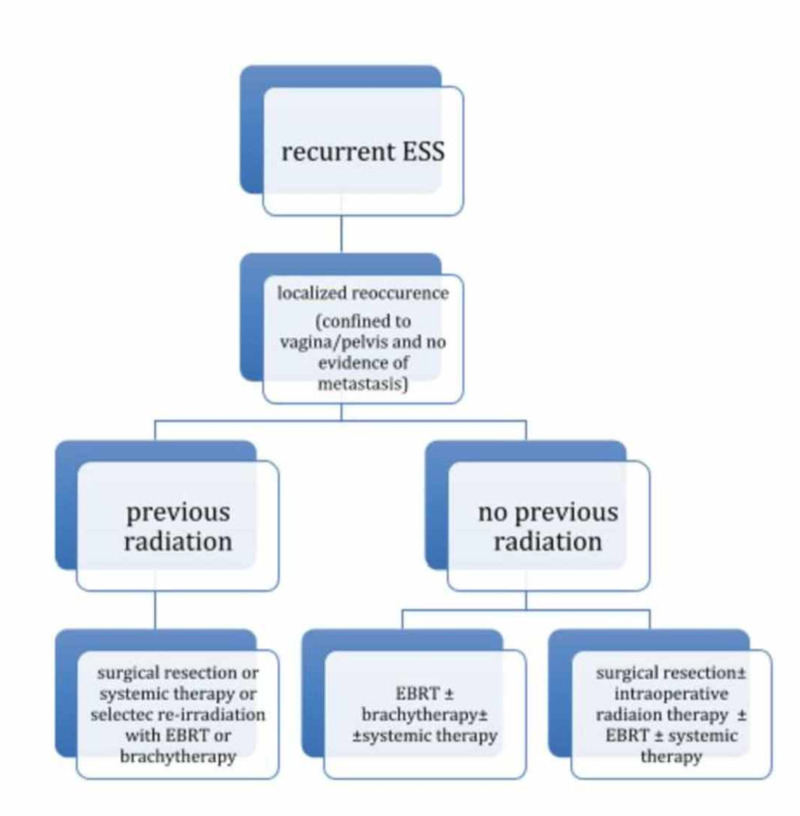
Continuation of treatment for recurrent ESS localized reocurrence

Treatment for recurrent ESS consists of the same modalities as in initial presentation but adapted to local recurrence, isolated metastasis, and disseminated disease. Another important characteristic in reappearance is to identify whether or not the patient had previous radiation therapy [10]. The role of adjuvant chemotherapy in ESS is still unclear as there is no survival benefit [2,13] (Figures 4-5).

Our patient underwent TAH and BSO for stage I LESS almost two decades ago in 1993. She neither received adjuvant EBRT or hormonal therapy, given the very early stage of the disease. She had a very long disease-free interval, as LESS is very slow-growing but was lost to follow-up. Due to the slow-growing nature of the disease, routine visits are an essential part of the patient's management.

## Conclusions

Uterine sarcomas are a heterogeneous and rare type of cancer that needs to be more extensively studied. Among uterine sarcomas, ESS deserves a special approach, as it can have a large pathological variation. The new nomenclature has helped with the meticulous classification of ESS as there are three clinicopathological entities. This classification has aided in determining prognosis and management but further research is needed in this area, as this patient population is scarce. There is also still a lot of ambiguity on how to manage recurrent ESS; in many cases, reoccurrence can be seen decades after a disease-free period. More randomized clinical trials are needed in order to study whether lifelong surveillance is needed or if there should be specific screening modalities.

## References

[1] Zagouri F, Dimopoulos AM, Fotiou S, Kouloulias V, Papadimitriou C (2009). Treatment of early uterine sarcomas: disentangling other adjuvant modalities. World J Surg Oncol.

[2] Gadducci A, Cosio S, Romanini A, Genazzani AR (2008). The management of patients with uterine sarcoma: a debated clinical challenge. Crit Rev Oncol Hematol.

[3] Puliyath G, Nair VR, Singh S (2010). Endometrial stromal sarcoma. Indian J Med Paediatr Oncol.

[4] (2020). American Cancer Society. Cancer statistics center. https://cancerstatisticscenter.cancer.org/.

[5] Zhang YY, Li Y, Qin M, Cai Y, Jin Y (2019). High-grade endometrial sarcoma: a retrospective study of factors influencing prognosis. Cancer Manag Res.

[6] Puliyath G, Nair MK (2012). Endometrial stromal sarcoma: A review of the literature. Indian J Med Paediatr Oncol.

[7] Alkasi O, Meinhold-Heerlein I, Zaki R, Fasching P, Maass N, Jonat W, Beckmann M (2009). Long-term disease-free survival after hormonal therapy of a patient with recurrent low grade endometrial stromal sarcoma: a case report. Arch Gynecol Obstet.

[8] D'Angelo E, Prat J (2010). Uterine sarcomas: a review. Gynecol Oncol.

[9] Li AJ, Giuntoli R, Drake R (2005). Ovarian preservation in stage I low grade endometrial stromal sarcoma. Obstet Gynecol.

[10] (2020). National comprehensive cancer network. Uterine sarcoma. https://www.nccn.org/framework/.

[11] Center of disease control and prevention (2020). Center of disease control and prevention: Uterine cancer: incidence and mortality-united states,1999-2016. Assessed on May 15, 2020.

[12] Chang KL, Crabtree GS, Lim-Tan SK, Kempson RL, Hendrickson MR (1993). Primary extrauterine endometrial stromal neoplasms: a clinicopathologic study of 20 cases and a review of the literature. Int J Gynecol Pathol.

[13] Li N, Wu LY, Zhang HT, An JS, Li XG, Ma SK (2008). Treatment options in stage I endometrial stromal sarcoma: a retrospective analysis of 53 cases. Gynecol Oncol.

[14] Spano JP, Soria JC, Kambouchner M, Piperno-Neuman S, Morin F, Morere JF, Martin A (2003). Long-term survival of patients given hormonal therapy for metastatic endometrial stromal sarcoma. Med Oncol.

[15] Yamazaki H, Todo Y, Mitsube K, Hareyama H, Shimada C, Kato H, Yamashiro K (2015). Long-term survival of patients with recurrent endometrial stromal sarcoma: a multicenter, observational study. J Gynecol Oncol.

[16] Chu MC, Mor G, Lim C, Zheng W, Parkash V, Schwartz PE (2003). Low-grade endometrial stromal sarcoma: hormonal aspects. Gynecol Oncol.

